# Placental Genetic Variants in the Upstream Region of the FLT1 Gene in Pre-eclampsia

**DOI:** 10.18502/jri.v21i4.4328

**Published:** 2020

**Authors:** Akiko Ohwaki, Haruki Nishizawa, Asuka Kato, Takema Kato, Jun Miyazaki, Hikari Yoshizawa, Yoshiteru Noda, Yoshiko Sakabe, Ryoko Ichikawa, Takao Sekiya, Takuma Fujii, Hiroki Kurahashi

**Affiliations:** 1- Department of Obstetrics and Gynecology, Fujita Health University School of Medicine, Toyoake, Japan; 2- Division of Molecular Genetics, Institute for Comprehensive Medical Science, Fujita Health University, Toyoake, Japan

**Keywords:** FLT1, Placenta, Pre-eclampsia, Short tandem repeat, Single nucleotide variant

## Abstract

**Background:**

Soluble fms-like tyrosine kinase 1 (sFlt-1) is believed to be a prominent component in the pathogenesis of pre-eclampsia, although the precise etiology has remained elusive. In this study, the etiological role of FLT1 variant was further validated in pre-eclampsia by examining this association in a Japanese sample population.

**Methods:**

The genotypes of three variants (rs4769613, rs12050029 and rs149427560) were examined in the upstream region of the FLT1 gene in placentas from pre-eclamptic (n=47) or normotensive control (n=49) pregnancy samples. Additionally, FLT1 mRNA levels in placenta were determined by qRT-PCR. ELISA was further used to detect circulating sFlt-1 levels in maternal sera. The intergroup comparisons were made using the Mann-Whitney U test or one way analysis of variance and P values of less than 0.05 were considered statistically significant.

**Results:**

First, the rs4769613 (C>T) and rs12050029 (G>A) genotypes were examined in placentas but no significant differences were found in the genotype or allele-type frequencies. Next, nearby short tandem repeat, rs149427560, was examined which manifested four size variants. In the genotypewise analysis, the frequency of the 474/476 heterozygote was significantly lower in pre-eclampsia (p<0.05). As expected, the FLT1 mRNA levels were significantly elevated in the pre-eclamptic placentas and sFlt-1 was higher in pre-eclamptic maternal sera. However, the genotype of these variants did not affect the FLT1 mRNA or serum sFlt-1 levels.

**Conclusion:**

Our findings did not support the hypothesis that genetic variations around the FLT1 gene affect the subtle expression changes underlying the etiologic pathway of pre-eclampsia. The hypothesis deserves further investigation through a larger sample size.

## Introduction

Pregnancy-induced hypertension, known as pre-eclampsia, is principally defined by the onset of hypertension with or without proteinuria (1), and is one of the most common obstetrical problems, accounting for almost 15% of pregnancy-associated disorders. Pre-eclampsia is not a simple complication of pregnancy but a multiple organ failure syndrome affecting the liver, kidneys, and lungs, in addition to coagulation and neural system. Although placentation and the maternal response are likely to be involved in the pathogenesis of pre-eclampsia, its precise etiology has remained elusive. It has been thought to be a polygenetic disorder, in which both fetal and maternal factors might be involved in its onset. A paradigm that has emerged is a two-stage disease hypothesis in which an initiating reduction of placental perfusion by abnormal vascular remodeling leads to the maternal symptoms in individuals who have a genetic predisposition to the disease (2). To date, a large number of genome-wide association studies or candidate gene approaches have been conducted and some candidate genes such as STOX1 and ACVR2A have been identified (3, 4). However, replicating these genetic associations in different populations has been challenging and hence no consistent genetic variants have been found that confer disease risk (5).

It has recently been reported that the genotype of the C/T variant near the FLT1 gene (rs4769613) in the offspring of a pre-eclamptic pregnancy is strongly associated with this disease (6). The encoded protein, sFlt-1, is a placenta-derived anti-angiogenic factor that is found at high concentrations in the maternal circulation in pre-eclampsia (7, 8). sFlt-1 acts as an anti-angiogenic factor by neutralizing proangiogenic factors such as VEGF (Vascular endothelial growth factor) or PlGF (Placental growth factor). As genetic variants associated with pre-eclampsia are located within the enhancer region of the FLT1 gene, it is not unreasonable to speculate that the minor allele enhances the transcription of the FLT1 gene and thereby facilitates the placental production of the sFlt-1 protein, leading to disease onset. The etiological role of FLT1 variant in pre-eclampsia was validated by examining this association in a Japanese sample population. The aim of this study was to confirm the hypothesis that genetic variations around the FLT1 gene affect the subtle expression changes underlying the etiologic pathway of pre-eclampsia.

## Methods

### Samples:

All of the clinical samples used in this study were collected at the Department of Obstetrics and Gynecology, Fujita Health University Hospital, Japan. Maternal blood and placental biopsy samples were obtained during caesarean sections from women with pre-eclampsia (n=47), and with a normal uncomplicated pregnancy (n=49). Pre-eclampsia was defined by a blood pressure of greater than 160/110 *mmHg*, and by proteinuria of more than 2 *g* in a 24 *hr* collection. Normotensive subjects were matched for maternal age and body mass index during pre-pregnancy. The clinical details of these subjects are presented in table 1.

**Table 1.** Characteristics of the study subjects with a normotensive pregnancy and with severe pre-eclampsia

	**Normotensive pregnancy (n=49)**	**Severe pre-eclampsia (n=47)**	**p-value**
**Gestational age (wks)**	35.1±4.7	33.3±3.3	p=0.035
**Maternal age (y)**	31.9±5.2	30.8±4.5	p=0.318
**Systolic BP (*mmHg*)**	113.3±9.5	165.4±13.3	p<0.001
**Diastolic BP (*mmHg*)**	67.3±9.8	102.6±12.3	p<0.001
Proteinuria ^*^	0 (0%)	45 (96%)	p<0.001
Body mass index (BMI) ^**^	22.0±4.1	22.0±4.2	p=0.967
**Birth weight (*g*)**	2434.8±860.5	1557.6±590.4	p<0.001
**Placental weight (*g*)**	525.2±145.7	320.2±91.3	p<0.001

# Data are given as the mean±standard deviation (SD).

* ≥2 *g* in a 24 *hr* collection.

** pre-pregnancy

To avoid any possible effects of labor on the FLT1 gene expression level, only placental samples that were obtained through caesarean section were included in the study cohort. A central area of chorionic tissue was then dissected, and the maternal decidua and amniotic membranes were removed (9). After vigorous washing of the maternal blood with saline, tissues were immediately frozen in liquid nitrogen and stored until use. Serum samples were stored at −80°*C* until use. Informed consent was obtained from each patient and this study was approved by the Ethical Review Board for Clinical Studies at Fujita Health University.

### Genotyping of the FLT1 gene:

Genomic DNA was extracted from the placentas using a commercially available kit in accordance with the manufacturer's protocol (Qiagen, Frankfurt, Germany). A total of 41 pre-eclampsia samples and 28 control samples from a normotensive pregnancy were used. TaqMan primers and probes were purchased to genotype the single nucleotide variants (SNVs) in the FLT1 gene (rs4769613, rs12050029) in accordance with the manufacturer’s protocol (C_32231378_10, C_1445411_10, Applied Biosystems, Foster City, CA). For the short tandem repeat (STR) variant (rs149427560), forward primers were labeled with FAM. The PCR products were analyzed by capillary electrophoresis (ABI3730 Genetic Analyzer; Applied Biosystems).

Genotype deviations from Hardy–Weinberg equilibrium (HWE) were first evaluated using the chi-square test. Genotype and allele frequency differences between the pre-eclamptic and control groups were then evaluated using chi-square analysis. All of these calculations were performed using SNPAlyze software (Dynacom, Chiba, Japan). Power calculations were performed using a genetic power calculator. To analyze the topologically associated domain (TAD) surrounding the FLT1 gene and the variants at that site, published Hi-C data obtained from the trophoblast cell line was used (http://promoter.bx.psu.edu/hi-c/view.php) (10, 11).

### Real-time RT–PCR:

To quantify FLT1 gene expression, real-time RT-PCR analyses were done using an ABI PRISM 7700 Sequence Detection System (Perkin-Elmer, Foster City, CA). Total RNA was extracted from chorionic villous tissue samples using RNeasy mini-kit (Qiagen, Valencia, CA), in accordance with the manufacturer's instructions. A Superscript First-strand Synthesis System for RT-PCR (Invitrogen, Grand Island, NY) using random primers was employed to produce single strand cDNA from the total RNA. PCR primers and TaqMan probes (Hs0105296_ *m1*) were obtained from Applied Biosystems GmbH (Weiterstadt, Germany). The housekeeping gene 18S rRNA (Hs99999901_s1) was used to normalize mRNA concentrations because many other genes that are commonly used as such a control are regulated by estrogen (12). RT-PCR reactions were performed in triplicate using a TaqMan EZ RT-PCR Kit (Perkin-Elmer) in a final volume of 25 *µl*. The conditions for RT were 5 *min* at 65*°C*, 30 *min* at 50*°C*, and 1 *min* at 95*°C*. The cycling conditions for PCR amplification were 30 *s* at 95*°C*, followed by 40 cycles of 5 *s* at 95*°C*, and 30 *s* at 60*°C*.

### Enzyme-linked immunosorbent assay (ELISA):

Serum sFlt-1 concentrations were measured using the Human VEGF R1/Flt-1 Quantikine ELISA Kit (DVR100B; R&D, Minneapolis, MN) in accordance with the manufacturer's instructions. A dilution series of recombinant human sFlt-1 was used to establish the standard curves. All samples were run in duplicate. The intra- and inter-assay coefficients of variation were less than 4.5% and 11.4%, respectively. The detection limit for recombinant sFlt-1 was approximately 10.0 *pg/ml*. The calibration was linear up to 1.0 *ng/ml*.

### Statistical analysis:

Genotype deviations from Hardy-Weinberg equilibrium were evaluated using the chi-square test (SAS/Genetics; SAS Japan, Inc., Tokyo, Japan). Marker-trait association analysis was used to evaluate allele- and genotype-wise associations with the chi-square test. For mRNA and protein analysis, intergroup comparisons were made using the Mann-Whitney U test or one way analysis of variance and p-values of less than 0.05 were considered statistically significant.

## Results

**Figure 1.**
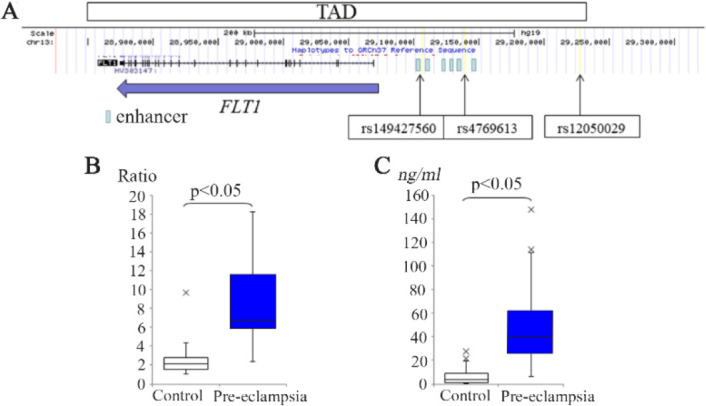
Genetic analysis of the FLT1 gene in pre-eclampsia. A) Location of three variants. The horizontal arrow indicates the location of the FLT1 gene exons and its transcription direction. The vertical arrows indicate the location of the three variants. The extent of the TAD including the FLT1 gene is indicated by a large box. Small boxes indicate the enhancer regions. B) Correlation between FLT1 gene expression in the placentas from pre-eclamptic and normotensive pregnancies. C) Correlation between the sFlt-1 levels in sera from pre-eclamptic and normotensive pregnancies. The boxes indicate the 25th and 75th percentiles. The bands near the middle indicate the median values. The bars indicate the 1.5 interquartile ranges with the outliers specifically marked

In this study, the association between pre-eclampsia and variations within the upstream region of the FLT1 gene (Figure 1A) was evaluated. First, rs4769613 and rs12050029 genotypes were examined, which were recently located within the different linkage disequilibrium blocks in their samples and found to be highly associated with pre-eclampsia (6). The genotypes in placentas from our pre-eclamptic or normotensive control pregnancy samples were detected but no significant differences in the genotype or allele-type frequencies were observed (Table 2). The genotype frequencies in our samples were comparable to those reported in East Asian populations, and the distribution satisfied the HWE.

**Table 2.** Association between placental SNVs near the FLT1 gene and severe pre-eclampsia

**SNV ID**		**Phenotype**	**MAF**	**N**	**Genotype**			**HWE**	**p-value**		
	
					***MM***	***Mm***	***mm***		**Genotype**		**Allele**

									**Dominant**	**Recessive**	
**SNV1**	rs4769613	Case	0.542	47	15	21	11	0.91	0.247	0.495	0.248
**C/T**		Control	0.448	49	10	24	15	1.0			

**SNV2**	rs12050029	Case	0.338	40	17	19	4	0.97	1.000	0.500	0.732
**G/A**		Control	0.303	38	17	19	2	0.7			

Next, another variant was examined in the upstream region of FLT1, rs149427560, which manifested size variations through different STR repeat numbers. Four size variants (472, 474, 476 and 478) were identified among our samples. By allelewise analysis, no significant difference was found in the allele-type frequency between the pre-eclamptic and normotensive control pregnancies (Table 3). In the genotypewise analysis however, the frequency of the 474/476 heterozygote was significantly lower in pre-eclampsia (p<0.05).

**Table 3.** Case-control study of the STR near the FLT1 gene in placentas from pre-eclamptic versus control pregnancies

		**Control**	**Pre-eclampsia**	**p-value**

**Sample number**		**43**	**47**	

		**(n=86)**	**(n=94)**	
**472**	4	2 (2.3%)	3 (3.2%)	p=0.724
**474**	5	23 (26.7%)	19 (20.2%)	p=0.365
**476**	6	54 (62.8%)	60 (63.8%)	p=0.885
**478**	7	7 (8.2%)	12 (12.8%)	p=0.313

**Genotype**		**(n=43)**	**(n=47)**	
**472/474**		0 (0.0%)	2 (4.3%)	p=0.171
**472/476**		2 (4.7%)	0 (0.0%)	p=0.135
**472/478**		0 (0.0%)	1 (2.1%)	p=0.336
**474/474**		4 (9.3%)	6 (12.8%)	p=0.554
**474/476**		14 (32.5%)	4 (8.5%)	p=0.004
**474/478**		1 (2.3%)	1 (2.1%)	p=0.949
**476/476**		16 (37.2%)	26 (55.3%)	p=0.054
**476/478**		6 (14.0%)	4 (8.5%)	p=0.412
**478/478**		0 (0.0%)	3 (6.4%)	p=0.090

As was expected from previously reported expression profiling data (9, 13), the FLT1 mRNA levels were found to be markedly higher in the placentas from our pre-eclampsia cases (Figure 1B). Accordingly, the serum sFlt-1 concentrations in the maternal circulation from our pre-eclampsia cases were also detected at significantly higher levels compared with the uncomplicated pregnancy subjects (Figure 1C).

**Figure 2.**
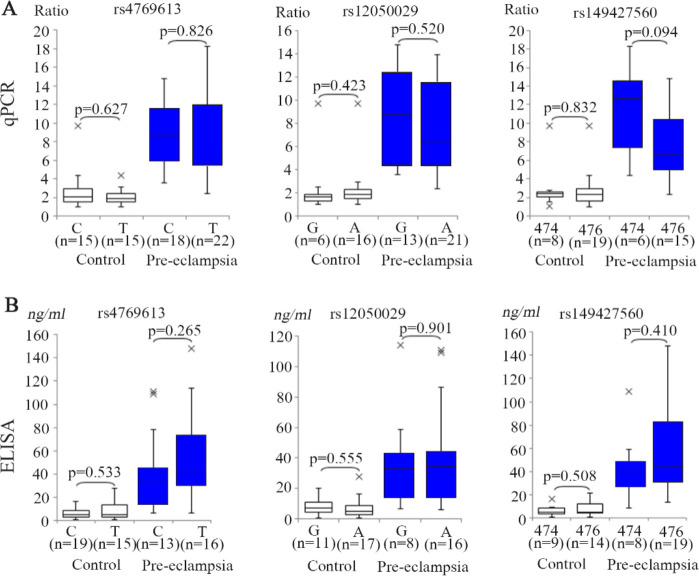
Analysis of the correlations between FLT1 gene expression, the serum concentrations of sFlt-1, and the three FLT1 variants. A) Comparison of the placental mRNA levels by variant allele-type. Data for rs4769613 (left), rs12050029 (center), and rs149427560 (Right) are shown. In each panel, data for the placentas from normotensive controls are shown on the left, and those from pre-eclamptic cases on the right. B) Comparison of the serum sFlt-1 levels by variant allele-type. The boxes indicate the 25th and 75th percentiles. The bands near the middle indicate the median values. The bars indicate the 1.5 interquartile ranges with the outliers specifically marked. Sample numbers and p-values are shown in each panel

Since the variants analyzed in this study are located within the TAD including the FLT1 gene (Figure 1A), it was hypothesized that they might affect the expression level of the FLT1 gene and thereby have an effect on disease susceptibility. A previous report analyzed the association between variations in the FLT1 gene and the gene expression levels using Genotype-Tissue Expression database but found no evidence of a correlation (6). The same analysis was concluded by qRT-PCR using our placental samples and no correlation was found between the genotypes or allele-types of the two SNVs and FLT1 gene expression (Figure 2). When the 474 and 476 alleles of the STR (rs149427560) were analyzed in this same way, again no correlation was found between the variant allele and the gene expression levels.

## Discussion

It has been well documented that pre-eclampsia is a multi-factorial disease that is induced by multiple genetic and environmental factors (14–16). With regard to the genetic factors involved, it is likely that both maternal and fetal factors affect the predisposition to pre-eclampsia. The recent "two-stage disease hypothesis" is that placental perfusion is initially reduced by abnormal vascular remodeling, which might be influenced by placental genetic factors (2). In its later stages, symptoms around the maternal circulation are the main component of this disease, in which maternal genetic factors might affect the genetic predisposition to its occurrence. Genetic factors in the maternal genome have been well investigated for this disorder. Although some maternal variants in the vasoactive genes have been consistently shown to increase the risk of pre-eclampsia (17), most of the genome wide association study (GWAS) that targeted the maternal genome have been unable to identify any variants associated with disease susceptibility. Further, exome sequencing of more than 8000 maternal genes has not identified any risk variants (18). It can be also envisaged, however, that variants in the placental genes may affect the onset of pre-eclampsia (19).

Genetic variations in the FLT1 gene (rs4769613) have recently been demonstrated by GWAS to be associated with a predisposition to pre-eclampsia using genomic DNA from offspring of a pre-eclamptic pregnancy (6). The increased production of anti-angiogenic factors, sFlt1 or endoglin, from trophoblast lineage cells is also known to be central to the abnormal vascular remodeling that leads to pre-eclampsia (7, 8, 20). There are now a large number of genetic association studies regarding vasoactive gene variants and pre-eclampsia, but the findings of these reports have not been reproducible in subsequent meta-analysis (5, 21). However, the association of FLT1 variants with pre-eclampsia has been replicated in several different ethnic populations from Europe (6, 22). Thus, the association between pre-eclampsia and the reported FLT1 SNVs in the Japanese population was examined, but no association was found in our pre-eclamptic placenta samples. However, this result may have been largely due to a low sample number that could not reach statistical significance.

It is notable that the rs149427560 STR has also been reported to be associated with pre-eclampsia (6), and has shown the significant genotype bias between pre-eclamptic placentas and controls. Within the TAD including the FLT1 gene, another previous report has demonstrated that an STR located in the 3’ untranslated region of the FLT1 gene was not associated with pre-eclampsia (23). However, a significant association was identified between this disease and the rs149427560 variant located within the placental enhancer region of FLT1 (Figure 1A) in this study. Our association analysis revealed that the 474/476 genotype was less frequent in the pre-eclampsia population, suggesting that this genotype acts as a protective allele.

To date, multiple lines of evidence have suggested that STRs affect the expression of nearby genes via the binding of transcription factors, the spacing between promotor elements, nucleosome packaging, histone modifications, or noncoding RNA functions (24– 26). It is not unreasonable to speculate therefore that upstream variations might affect the expression of the FLT1 gene leading to a stronger predisposition to pre-eclampsia. The expression of the FLT1 gene was examined at the mRNA and protein levels since the variations are located in the placental enhancer region, but the allele-type did not correlate with the gene expression levels. One possible explanation for this is that the difference in the expression was below the threshold required to detect statistical significance. The small sample size might have impacted upon our findings. The difference in FLT1 expression levels between pre-eclamptic placentas and normotensive controls is considerable, possibly because of the synergistic effect of multiple genetic factors including these variants. On the other hand, it is also possible that elevated serum sFlt-1 levels are the result of a secondary response to some other factors that initially trigger the onset of pre-eclampsia.

## Conclusion

Our present findings regarding the association of a nearby STR upstream of the FLT1 gene with pre-eclampsia may indicate that genetic variations in this gene underlie the predisposition to pre-eclampsia. The hypothesis that genetic variations around the FLT1 gene affect the subtle expression changes underlying the etiologic pathway of pre-eclampsia deserves further investigation through a large scale study with appropriate number of samples.
